# Supplementary material for: A stalled-ribosome rescue factor Pth3 is required for mitochondrial translation against antibiotics in Saccharomyces cerevisiae
Source: Commun Biol. 2021 Mar 8;4:300. doi: 10.1038/s42003-021-01835-6 (PMC7940416; doi:10.1038/s42003-021-01835-6)
Supplement: Supplementary file 2 — Supplementary Information [file 42003_2021_1835_MOESM2_ESM.pdf]

Supplementary Information

A stalled-ribosome rescue factor Pth3 is required for mitochondrial translation against antibiotics in *Saccharomyces cerevisiae*.

Soichiro Hoshino, Ryohei Kanemura, Daisuke Kurita, Yukihiro Soutome, Hyouta Himeno, Masak Takaine, Masakatsu Watanabe, and Nobukazu Nameki

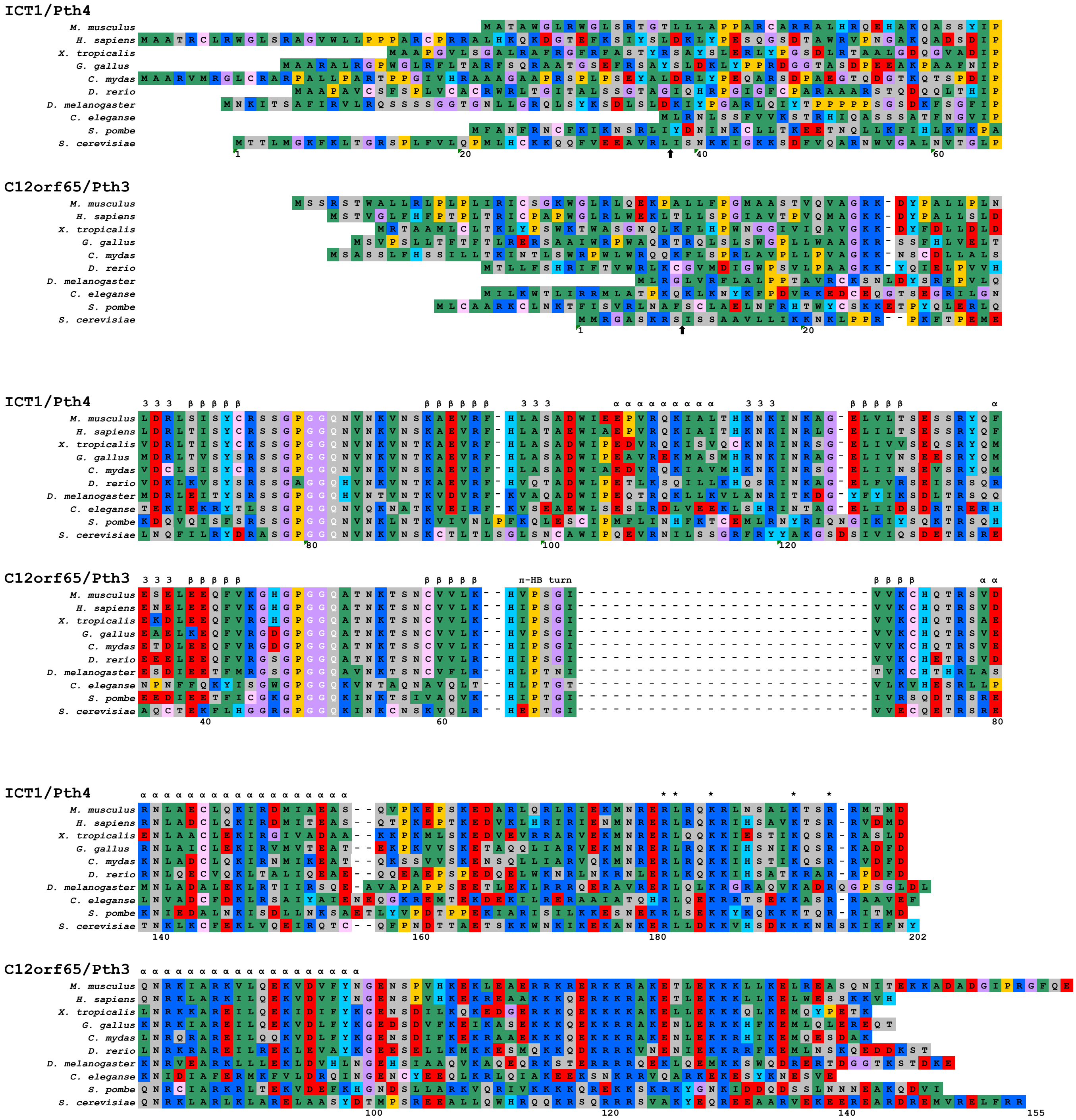

Supplementary Figure 1. Sequence alignment of ICT1/Pth4 and C12orf65/Pth3 homologs from eukaryotes. Secondary structural elements of ICT1 (PDB ID 1J26) and C12orf65 (2RSM) from *Mus musculus* are indicated. The method and accession codes used in the sequence alignment, except for the codes as below, are previously described[1]: *Chelonia. mydas* ICT1, XP_007065098.1; *C. mydas* C12orf65, XP_007071680.1; *Danio. rerio* ICT1, NP_001313644.1; *D. rerio* C12orf65, XP_001340041.2; *S. pombe* Pth4, NP_594059.1; *S. pombe* Pth3, NP_596474.2. The truncation positions for Pth4 and Pth3 from *S. cerevisiae* used in recombinant His-tagged proteins are marked by vertical arrows. White characters indicate the conserved GGQ motif. Asterisks indicate residues required for PTH activity that were shown in the mutational analysis using YaeJ and ICT1[2]. Alignments are colored as follows: purple: glycine (G); yellow: proline (P); green: small and hydrophobic amino acids (A, V, L, I, M, F, and W); gray: hydroxyl and amide amino acids (S, T, N, and Q); red: negatively charged amino acids (D and E); blue: positively charged amino acids (K and R); pink: cysteine (C); cyan: histidine (H) and tyrosine (Y).

YPD, 30 °C, 24 h

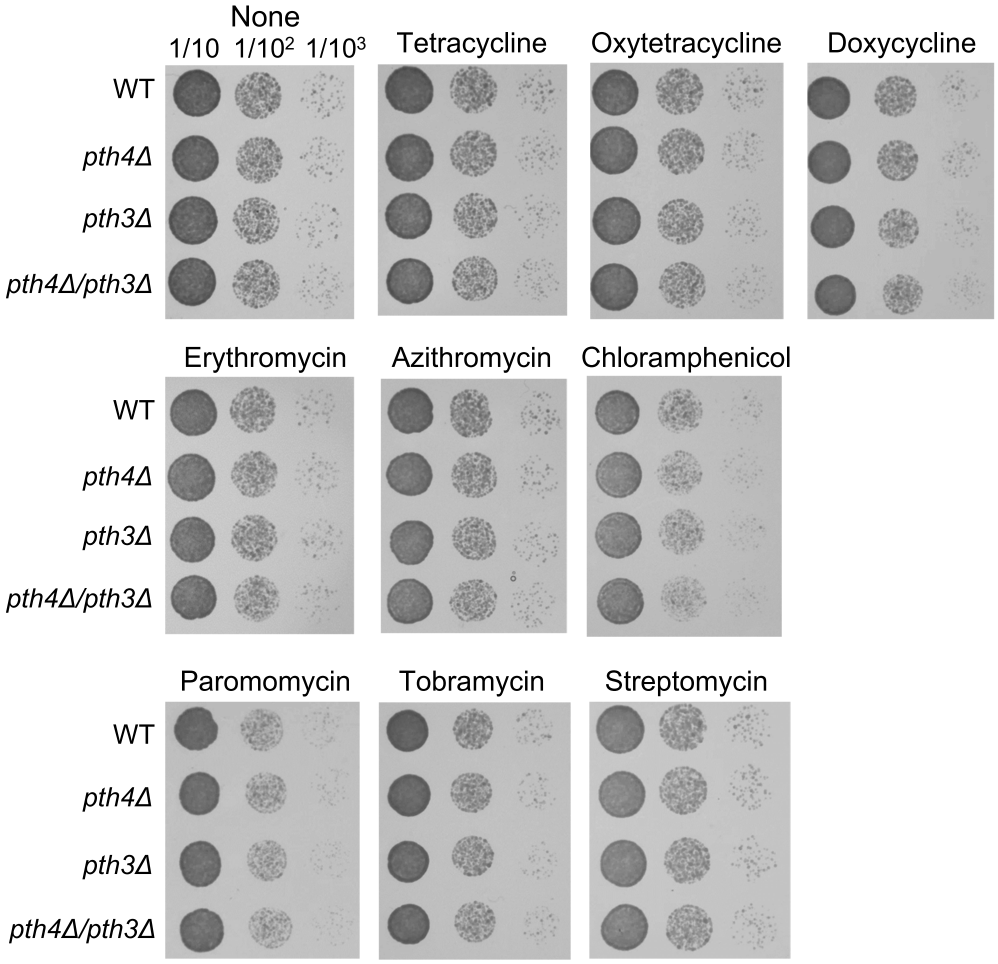

Supplementary Figure 2. No susceptibility to antibiotics by gene deletion mutants of *S. cerevisiae* grown on YPD plates containing glucose as a carbon source.

Dilutions of the wild type (WT), *pth4*Δ, *pth3*Δ, and *pth4*Δ*pth3*Δ were spotted onto YPD plates including each antibiotic. Dilution rates were larger than those in YPG media (Fig. 1) to clarify the effect of each antibiotic on growth in YPD media. Plates were incubated at 30 °C for 24 h.

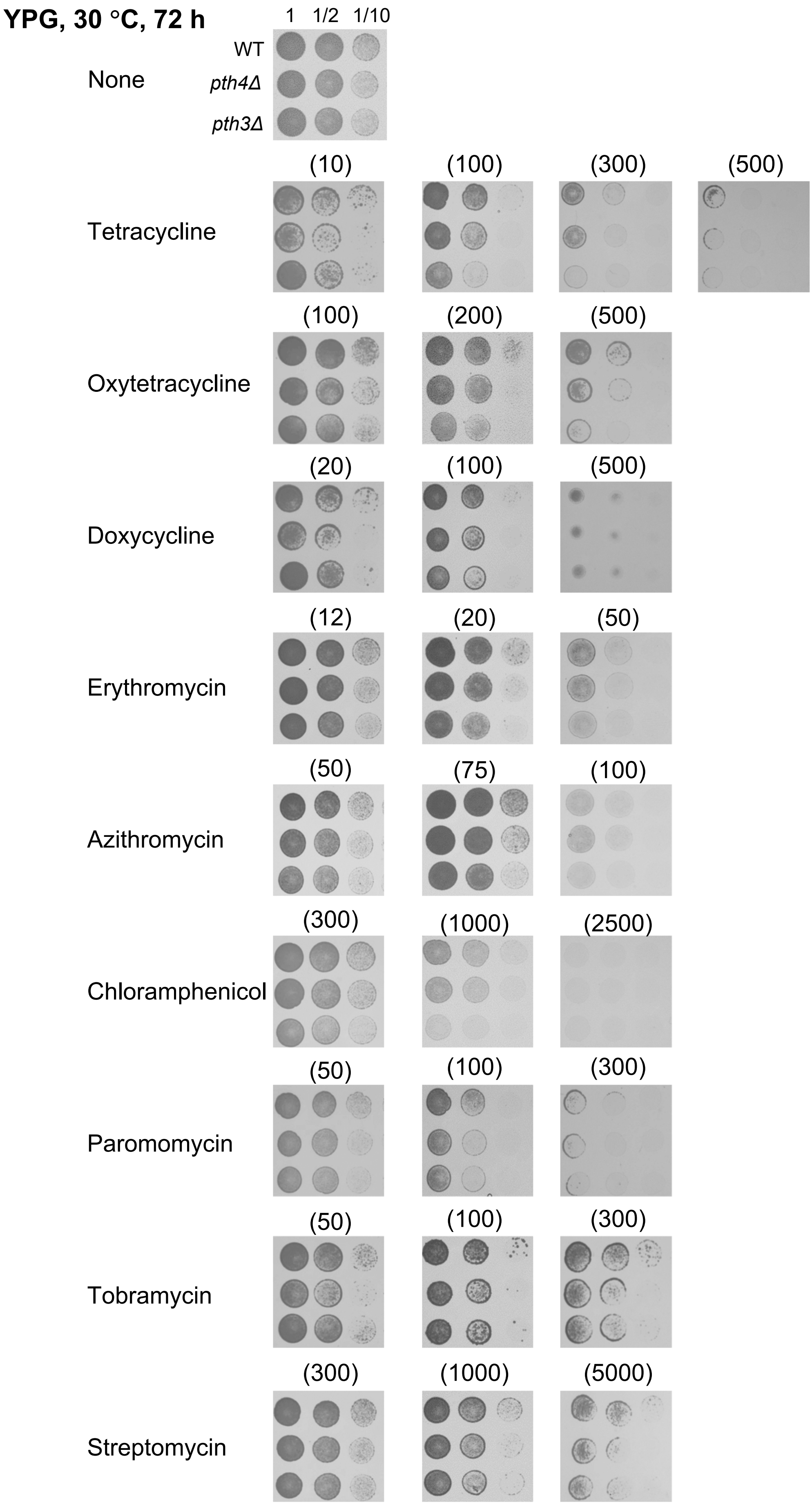

Supplementary Figure 3. Concentration-dependent susceptibility to antibiotics by the gene deletion mutants of *S. cerevisiae* on YPG plates.
Each concentration is indicated in parentheses (µg/mL). Plates were incubated at 30 °C for 72 h.

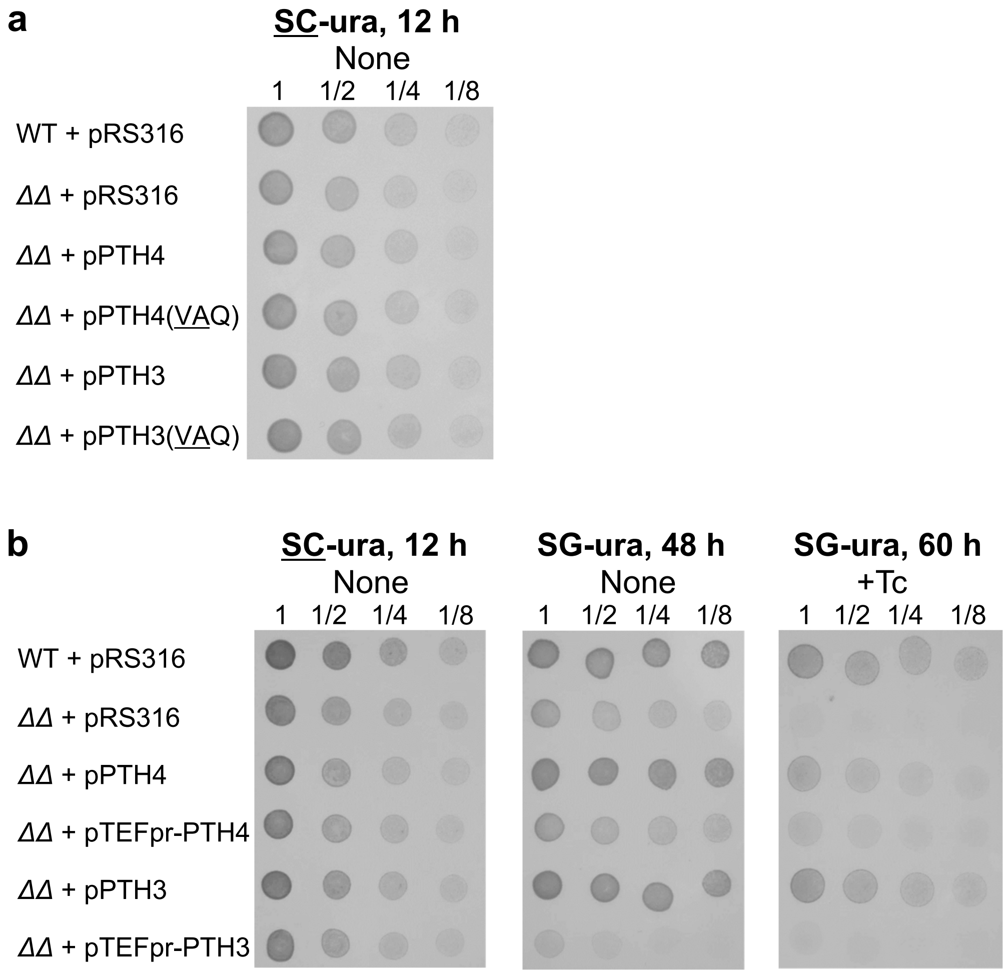

Supplementary Figure 4. Suppression of the antibiotic-susceptibility phenotype of the double-gene deletion mutant by plasmid-borne *PTH3* or *PTH4* using a TEF1 promoter instead of a native promoter.

(a) The six strains shown in Fig. 2 were spotted onto SC-ura plates containing glucose in the absence of an antibiotic.

(b) The native promoter in plasmids pPTH4 and pPTH3 was replaced with a strong constitutive TEF1 promoter to yield plasmids pTEFpr-PTH4 and pTEFpr-PTH3, respectively. The *pth4*Δ*pth3*Δ mutant (represented by ΔΔ) was transformed with pPTH4, pTEFpr-PTH4, pPTH3, pTEFpr-PTH3 or an empty plasmid pR316, and the resultant strains were diluted and spotted onto SC-ura plates, and SG-ura plates in the absence or presence of 100 μg/mL tetracycline (Tc).

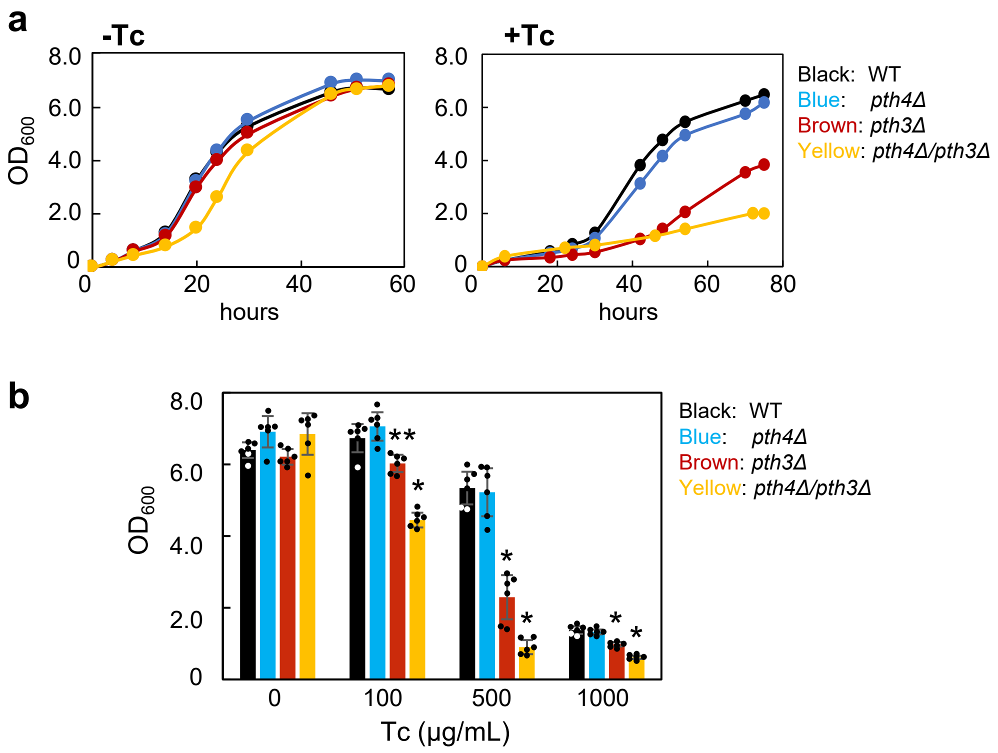

Supplementary Figure 5. Effects of tetracycline on growth of *pth4*Δ, *pth3*Δ, and *pth4*Δ*pth3*Δ grown in liquid YPG media.

(a) An example of obtained growth curves of the three mutants and the wild type. They are inoculated in liquid YPG media in the absence or presence of 500 µg/mL tetracycline (Tc) with a starting OD_{600} of 0.01, and growth was monitored at 30 °C. (b) Dependence of Tc concentrations on growth of the three mutants and the wild type. Shown were OD_{600} values of each strain grown for 48 h in liquid YPG media containing Tc at the indicated concentrations. Data are presented as the mean ± standard deviation of six independent experiments. Asterisks indicate significant difference from the wild type (Student's t test, $^*P < 0.001$ and $^{**}P < 0.01$).

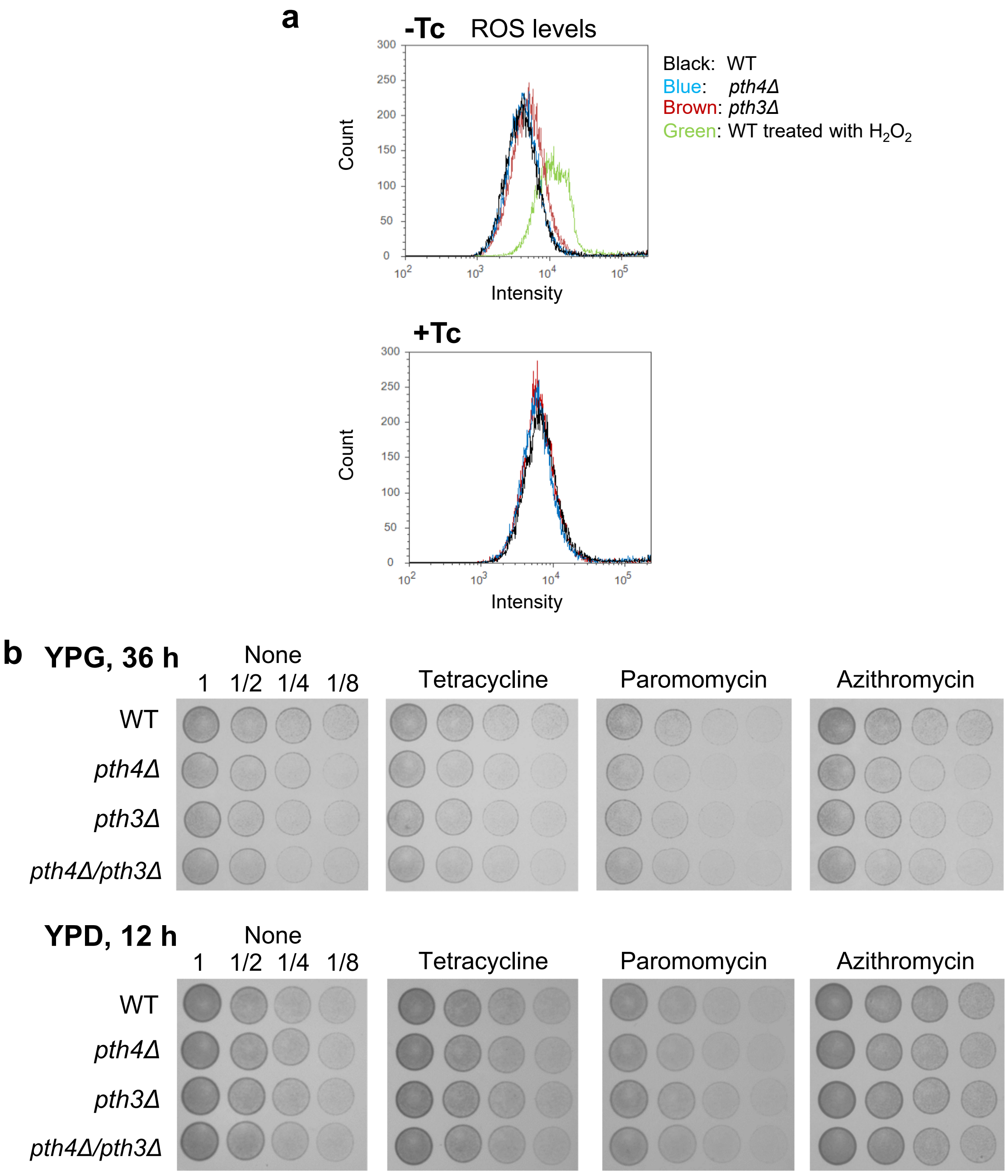

Supplementary Figure 6. A small effect of tetracycline on ROS generation in *pth4*Δ and *pth3*Δ, and growth arrest of the mutants induced by tetracycline, paromomycin or azithromycin.

(a) ROS levels in the wild type (WT), *pth4*Δ, and *pth3*Δ grown in liquid YPG media for 24 h were measured by FCM using DCFH-DA in the absence or presence of 500 μg/mL tetracycline (Tc). H_2O_2 was used as a ROS generator. (b) The mutants and the wild type were grown for 24 h in YPG liquid media in the absence of any antibiotics. They were harvested and resuspended in YPG media containing 1 mg/mL tetracycline, 1 mg/mL paromomycin or 0.75 mg/mL azithromycin. After incubation at 30 °C for 1 h, they were harvested and washed with PBS buffer to remove each antibiotic. The resultant samples were spotted on YPG or YPD plates in the absence of any antibiotics.

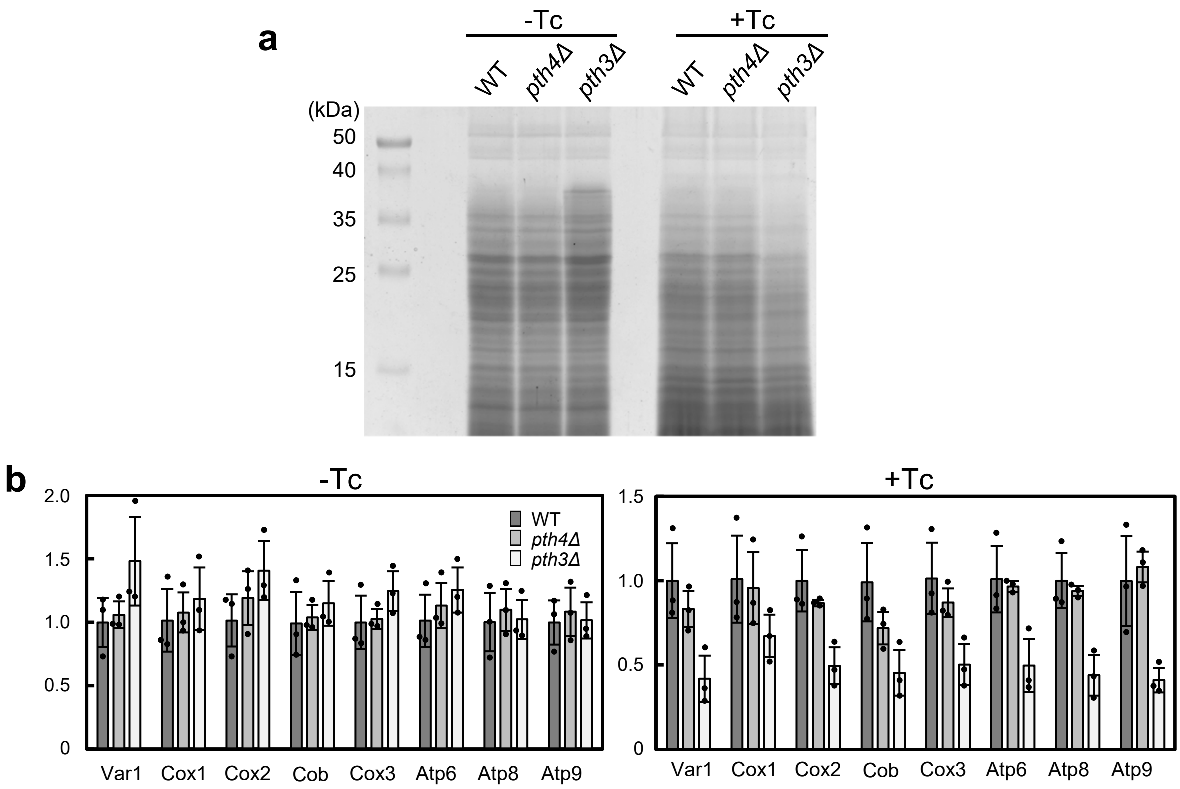

P-values	Var1	Cox1	Cox2	Cob	Cox3	Atp6	Atp8	Atp9	Var1	Cox1	Cox2	Cob	Cox3	Atp6	Atp8	Atp9
WT vs *pth4Δ*	0.83	0.79	0.67	0.88	0.91	0.75	0.74	0.90	0.35	0.53	0.091	0.30	0.59	0.74	0.17	0.58
WT vs *pth3Δ*	0.39	0.66	0.39	0.73	0.70	0.64	0.94	0.94	**0.058**	0.29	**0.067**	**0.10**	**0.094**	0.16	**0.039**	0.14

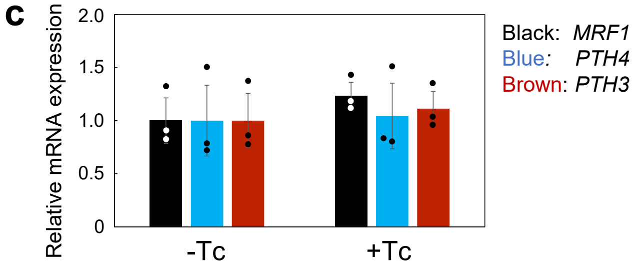

Supplementary Figure 7. Effects of the deletion of *PTH4* or *PTH3* on mitochondrial translation in the absence or presence of tetracycline and those of tetracycline on mRNA expression of *PTH4* or *PTH3* in the wild type.

(a) Coomassie blue staining of SDS-PAGE gels showing equal amounts of total proteins among *pth4*Δ, *pth3*Δ, and the wild type. Mitochondria lysate samples corresponding to those used in radioisotope labeling (Fig 4a) were subject to SDS-PAGE followed by Coomassie blue staining. (b) Quantification of mitochondrial translation products synthesized in organelles from the wild type, *pth4*Δ, and *pth3*Δ grown in YPG media for 24 h in the absence or presence of 500 μg/mL tetracycline (Tc). Band intensities corresponding to the eight proteins in the gel as shown in Fig. 4a were quantified using ImageQuant software. Data are shown as the mean ± standard deviation of three independent experiments. P-values are shown in the tables. Bold values are significant at the 0.1 level. (c) qPCR data of *PTH4*, *PTH3* and *MRF1* from total RNAs extracted from the wild type grown for 48 h in liquid YPG media in the absence or presence of 500 μg/mL Tc. *MRF1* codes for mitochondrial release factor (mtRF1) (Table 1). Data are presented as the mean ± standard deviation of three independent experiments.

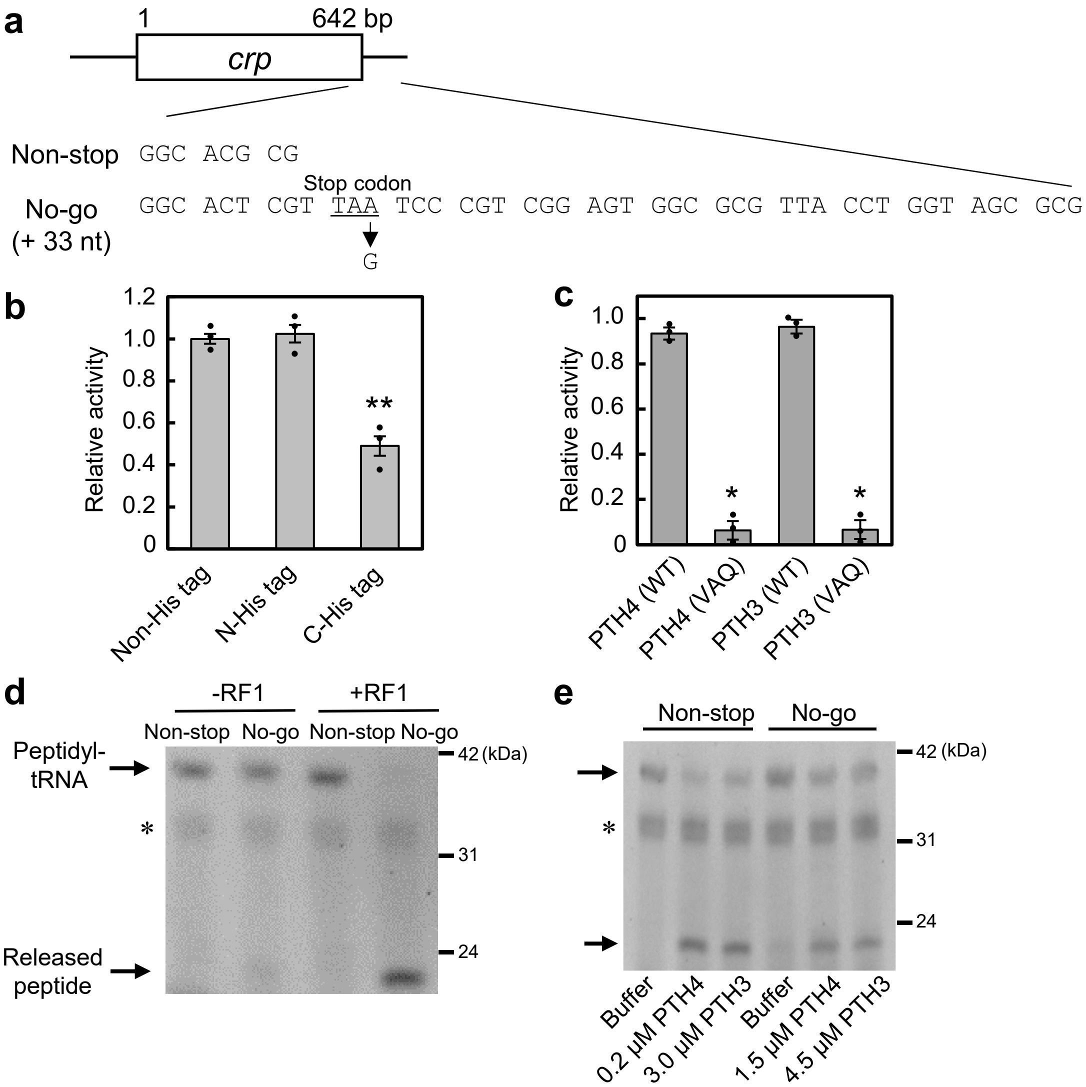

Supplementary Figure 8. *In vitro* translation of the non-stop or no-go template with some versions of recombinant His-tagged Pth3 and Pth4 proteins.

(a) Schematic drawing of non-stop and no-go templates, which were used in the *in vitro* translation system. The box indicates the open reading frame of the *crp* gene. In the no-go template, the stop codon, TAA, was changed to another stop codon, TA<u>G</u>, which is only recognized by RF1. (b) Comparison of PTH activities toward non-stop ribosomes among three recombinant Pth3 proteins, each of which contains a His-tag at the N or C terminus or no His-tag. The non-His-tagged protein was obtained by the His-tag being removed from the N-terminal-tagged protein using thrombin. The final concentration of the three proteins was 3.0 μM and the other experimental conditions are described in the legend of Fig. 5a. Data are presented as the mean ± standard deviation of three independent experiments. Asterisks indicate significant difference from the wild type (Student's t test, *P < 0.001 and **P < 0.05). (c) *In vitro* translation of the non-stop template with the Pth3 and Pth4 mutants in which the GGQ residues were changed to VAQ. Details are described in the legend of Fig. 5a. The concentrations of the Pth4 and Pth3 proteins were 0.45 and 9.0 μM, respectively. (d) *In vitro* translation of the non-stop or no-go template in the absence or presence of RF1. The addition of 3.0 μM RF1 only shifted the band indicating peptidyl-tRNAs on no-go ribosomes to a band showing released peptides. An asterisk indicates a background band (approximately 30 kDa) that appears without the addition of template DNA or mRNA, as previously shown in the manufacturer's technical note. These results showed that no-go ribosomes stalled at a stop codon in the decoding site, as shown in Fig. 5b. (e) An example of gel images for comparison of concentrations between Pth4 and Pth3 proteins that were required for approximately 60% PTH activities toward non-stop or no-go ribosomes. Details are described in Fig. 5b.

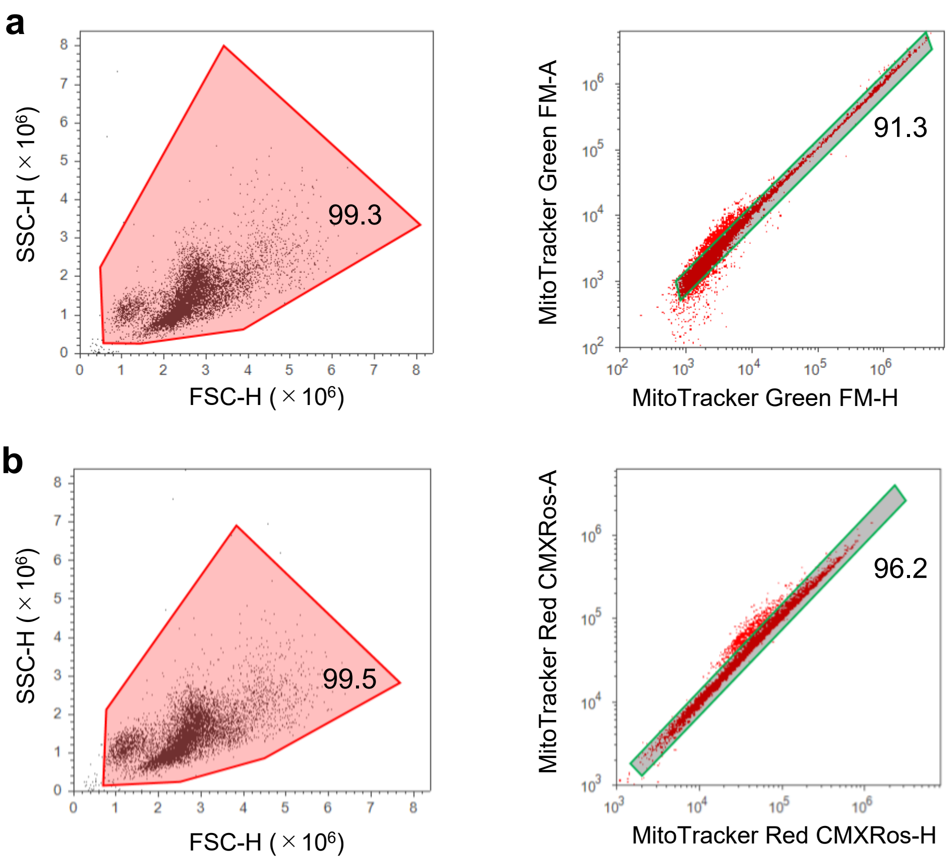

Supplementary Figure 9. Flow cytometry gating strategy for the experiments shown in Figure 3b.

For examples, the wild-type cells grown in YPG medium for 24 h were stained with MitoTracker Green FM (a) or MitoTracker Red CMXRos (b). A total of 20,000 cells per sample were measured. Debris was removed in FSC-H versus SSC-H plots (left panels), and then doublets were excluded based on area versus height plots of each MitoTracker signal (right panels).

Fig. 4a

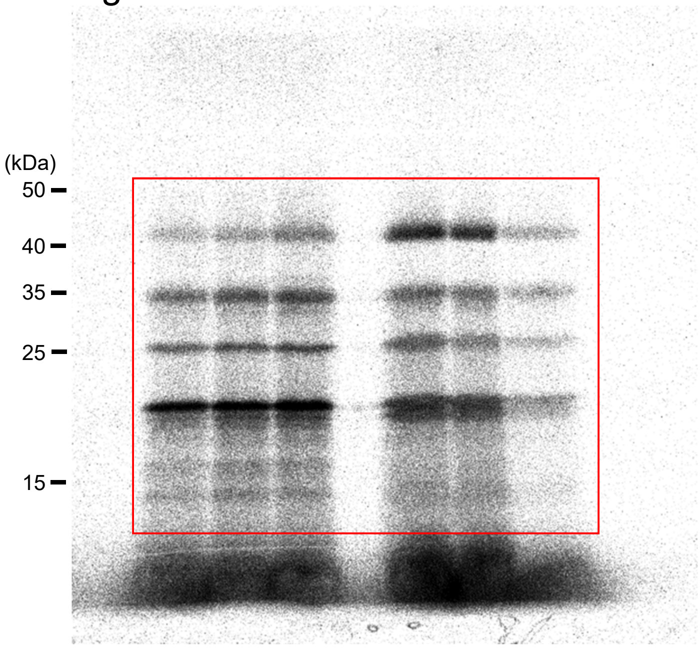

Fig. 5a **Pth4**

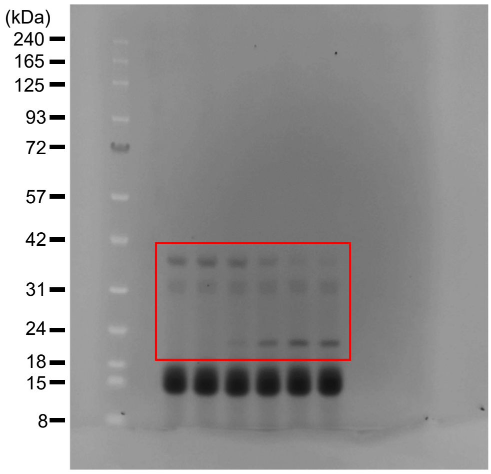

Pth3

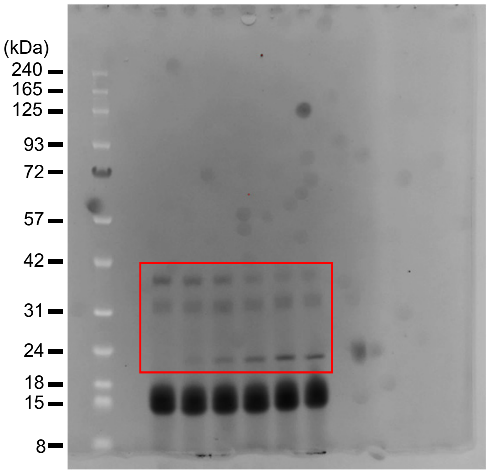

Supplementary Fig. 8d

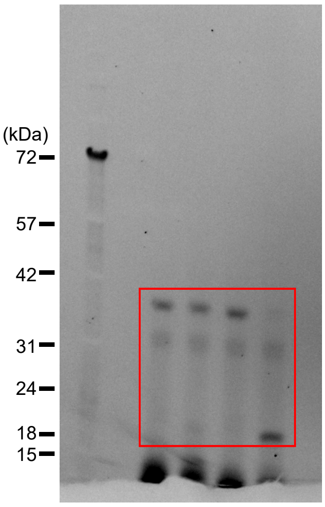

Supplementary Fig. 8e

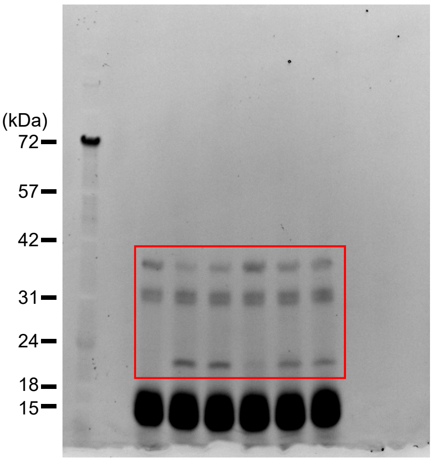

Supplementary Figure 10. Uncropped gel images for the indicated figures.

Red boxes show the cropped regions.

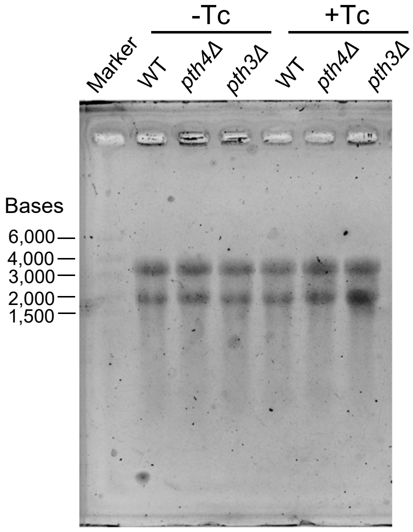

Supplementary Figure 11. Agarose gel electrophoresis for RNA quality check on total RNA extracted from the wild type, *pth4*Δ and *pth3*Δ grown in different conditions.

The wild type, *pth4*Δ and *pth3*Δ were grown for 24 h in YPG media or for 48 h in YPG media in the absence or presence of 500 µg/mL tetracycline (Tc). 8 µg of total RNA of each sample was loaded onto a 1% formaldehyde denaturing agarose gel. Each sample shows clear 26S and 18S rRNA bands. The RNA samples were used in qPCR experiments.

Supplementary Table 1. Summary of properties of ribosome-binding antibiotics used in the present study.

Class	Antibiotic	Target[1)]	Inhibition mechanism[2)]	Yeast mito-chondria[3)]	Human mito-chondria[3)]
Tetracyclines	Tetracycline	S	tRNA delivery	3	4, 5
	Oxytetracycline	S	tRNA delivery	3	6, 7
	Doxycycline	S	tRNA delivery	–	5, 6
Macrolides	Erythromycin	L	Nascent chain elongation	3, 8, 9	4
	Azithromycin	L	Nascent chain elongation	–	10
Amphenicols	Chloramphenicol	L	Peptidyl-transferase center	3, 8, 9	4, 11
Aminoglycosides	Paromomycin	S	Translocation	12-14	15
	Tobramycin	S	Translation elongation	–	15
	Streptomycin	S	Translocation	16	17

1, 2) These parts of this table were prepared by referring to two reviews[18, 19].

1) S and L indicate the small and large ribosomal subunits, respectively.

3) These references indicate that the antibiotic affects mitochondrial translation or at least causes mitochondrial dysfunction in yeast or humans.

Supplementary Table 2. Strains used in this study.

Name	Genotype	Source
MTY3015	*his3Δ1 leu2Δ0 lys2Δ0 ura3Δ0 Mat a*	Lab stock
MTY3016	*his3Δ1 leu2Δ0 lys2Δ0 ura3Δ0 Mat α*	Lab stock
MTY3462	*yol114cΔ::HIS3 his3Δ1 leu2Δ0 lys2Δ0 ura3Δ0 Mat a*	This study
MTY3458	*yol114cΔ::HIS3 his3Δ1 leu2Δ0 lys2Δ0 ura3Δ0 Mat α*	This study
MTY3459	*ylr281cΔ::HIS3 his3Δ1 leu2Δ0 lys2Δ0 ura3Δ0 Mat α*	This study
MTY3464	*yol114cΔ::HIS3 ylr281cΔ::HIS3 his3Δ1 leu2Δ0 lys2Δ0 ura3Δ0 Mat α*	This study
BL21(DE3) RIPL	*F- ompT hsdS (rB-mB-) dcm Tetr gal λ(DE3) endA Hte [argU ileY leuW proL* Cam^r*]*	Agilent Technologies

Supplementary Table 3. Plasmids used in this study.

Name	Structure	Source
MTP0097	pFA6a-His3MX6	Lab stock
MTP3087	pMitoLOC	Lab stock; Addgene (cat. # 58980)
MTP0094	pRS316	Lab stock
pPTH4	pRS316-$P_{YOL114C}$-YOL114C/PTH4-$T_{YOL114C}$	This study
pPTH3	pRS316-$P_{YLR281C}$-YLR281C/PTH3-$T_{YLR281C}$	This study
pPTH4(VAQ)	pRS316-$P_{YOL114C}$-YOL114C(VAQ)-$T_{YOL114C}$	This study
pPTH3(VAQ)	pRS316-$P_{YLR281C}$-YLR281C(VAQ)-$T_{YLR281C}$	This study
pET15b	T7 RNA polymerase expression plasmid encoding N-terminal His6 peptide epitope, Amp^r	Novagen
pET26b	T7 RNA polymerase expression plasmid encoding C-terminal His6 peptide epitope, Kan^r	Novagen
pET15b 114C	Encoding the YOL114C (Δ1-37), Amp^r	This study
pET26b 114C	Encoding the YOL114C (Δ1-37), Kan^r	This study
pET15b 281C	Encoding the YLR281C (Δ1-9), Amp^r	This study
pET26b 281C	Encoding the YLR281C (Δ1-9), Kan^r	This study
pET26b 114C VAQ	Encoding the YOL114C (Δ1-37) VAQ, Kan^r	This study
pET15b 281C VAQ	Encoding the YLR281C (Δ1-9) VAQ, Amp^r	This study

Supplementary Table 4. Primers used in this study.

Name	Sequence (5′ → 3′)	Purpose
pFA6a-His3MX6 114C primer (F)	5′- TACTGTTTGCCTTTCAGTAAATTG<br>AAGGGAAACACGCCAAGAAATATCATcgga<br>tccccgggttaattaa -3′	Preparation of YOL114C/PTH4-deficient strain
pFA6a-His3MX6 114C primer (R)	5′- CATCACTGTGTACCTGGATAGAAT<br>GTGTGTTTAAATATGCGTATTATGTA<br>gaattcgagctcgtttaaac -3′	
pFA6a-His3MX6 281C primer (F)	5′- AAGCTATGCGACCCTGGAAAAAGC<br>GCAGAAGAATCCGAGACTAGTAAACT<br>cggatccccgggttaattaa -3′	Preparation of YLR281C/PTH3-deficient strain
pFA6a-His3MX6 281C primer (R)	5′- TTTTTTTTCATTTTTTTTTTCTTTTC<br>TCCTCCATCTAATTTCACCTGCGG<br>gaattcgagctcgtttaaac -3′	
pRS316 114C genomic (F)	5′- tggcggccgctctagAAGGCACAG<br>ATAAGACTGGTTCC -3′	Preparation of pPTH4
pRS316 114C genomic (R)	5′- cgggccccccctcgaTCGTGGAAA<br>GGCAAGAGATTT -3′	
pRS316 281C genomic (F)	5′- tggcggccgctctagCGGAAAAAT<br>TGCCTCTTTCA -3′	Preparation of pPTH3
pRS316 281C genomic (R)	5′- cgggccccccctcgaTGTTAATGG<br>AACGACCGATCT -3′	
pRS316 114C VAQ (F)	5′- gttgctcaaAACGTTAATAAGG<br>TAAATAG -3′	Preparation of pRS316 and pET harboring PTH4(VAQ)
pRS316 114C VAQ (R)	5′- TGGCCCGCTGGCTCTATC -3′	
pRS316 281C VAQ (F)	5′- GGCCCgttgctcaaAAGATTAA<br>TAAGTGTAATTCTAAGG -3′	Preparation of pRS316 and pET harboring PTH3(VAQ)
pRS316 281C VAQ (R)	5′- CTCTGCCGCCATGAAGG -3′	
pET15b Δ37 YOL114C (F)	5′- cgcggcagccatatgATTAGTAATAA<br>AAAGATCGGAAAG -3′	Preparation of pET15b 114C
pET15b Δ37 YOL114C (R)	5′- gttagcagccggatccTTAATAATTGA<br>ATTTAATTTTACTTCTG -3′	
pET26b Δ37 YOL114C (F)	5′- ggagatatacatatgATTAGTAATAA<br>AAAGATCGGAAAG -3′	Preparation of pET26b 114C/114C(VAQ)
pET26b Δ37 YOL114C (R)	5′- gtggtggtgctcgagATAATTGAATTT<br>AATTTTACTTCTG -3′	

pET15b Δ9 YLR281C (F)	5′- cgcggcagccatatgATCAACAGTGC CGCAGTGCTGCTG -3′	Preparation of pET15b 281C/281C(VAQ)
pET15b Δ9 YLR281C (R)	5′- gttagcagccggatccTTACCGGCGGA ATAACTCGCGCAC -3′	
pET26b Δ9 YLR281C (F)	5′- ggagatatacatatgATCAACAGTGCC GCAGTGCTGCTG -3′	Preparation of pET26b 281C
pET26b Δ9 YLR281C (R)	5′- gtggtggtgctcgagCCGGCGGAATAA CTCGCGCAC -3′	

Lower-case letters indicate sequence homologs to that in the plasmid for In-Fusion cloning.

Supplementary Table 5. Primers used in qPCR experiments.

Name	Sequence (5′ → 3′)	Purpose
Act1_qPCR primer (F)	5′- ACGGTATTGTCACCAACTGGGACG -3′	Reference of qPCR
Act1_qPCR primer (R)	5′- ACAGGGTGTTCTTCTGGGGCAA -3′	
Cox1_qPCR primer (F)	5′- CTACAGATACAGCATTTCCAAGA -3′	mRNA of *COX1*
Cox1_qPCR primer (R)	5′- GTGCCTGAATAGATGATAATGGT -3′	
Cox2_qPCR primer (F)	5′- AGATTCGTTGTAACAGCTGCTGA -3′	mRNA of *COX2*
Cox2_qPCR primer (R)	5′- TCTACCAGGAGTAGCATCAACTTTA -3′	
Cox3_qPCR primer (F)	5′- TGAAGCTGTACAACCTACCGA -3′	mRNA of *COX3*
Cox3_qPCR primer (R)	5′- ACCTGCGATTAAGGCATGATGA -3′	
Cob_qPCR primer (F)	5′- TGTATCTTGATTATGAGGTGGGT -3′	mRNA of *COB*
Cob_qPCR primer (R)	5′- CCTAATGGATTAGATGAACCATG -3′	
Atp6_qPCR primer (F)	5′- ATTCGTACCTGCTGGTACAC -3′	mRNA of *ATP6*
Atp6_qPCR primer (R)	5′- ATGACCAGCTAAGATATTAGAACC -3′	
Atp8_qPCR primer (F)	5′- CACAATTAGTTCCATTTTATTTTATG -3′	mRNA of *ATP8*
Atp8_qPCR primer (R)	5′- GATCATAGGTAAAAAGAATTGTGAG -3′	
Atp9_qPCR primer (F)	5′- GGAGCAGGTATTGGTATTGC -3′	mRNA of *ATP9*
Atp9_qPCR primer (R)	5′- AGCTTCTGATAAGGCGAAAC -3′	
Yol114c_qPCR primer (F)	5′- GGTAGGAGCACTTAACGTAACG -3′	mRNA of *PTH4*
Yol114c_qPCR primer (R)	5′- CCTGAGGAATCCAAGCAC -3′	
Ylr281c_qPCR primer (F)	5′- GAGTAAGAGGTCAATCAGCAGTG -3′	mRNA of *PTH3*
Ylr281c_qPCR primer (R)	5′- AGTGCATTGTGCTTCCATC -3′	
MRF1_qPCR primer (F)	5′- GCATTATCGATGCCATTCT -3′	mRNA of *MRF1*
MRF1_qPCR primer (R)	5′- CAGTGGATGTGTGCGTC -3′	

Supplementary References

1. Kogure, H. *et al.* Solution structure and siRNA-mediated knockdown analysis of the mitochondrial disease-related protein C12orf65. *Proteins* **80**, 2629-2642 (2012).
2. Kogure, H. *et al.* Identification of residues required for stalled-ribosome rescue in the codon-independent release factor YaeJ. *Nucleic Acids Res.* **42**, 3152-3163 (2014).
3. Clark-Walker, G.D. & Linnane, A.W. *In vivo* differentiation of yeast cytoplasmic and mitochondrial protein synthesis with antibiotics. *Biochem. Biophys. Res. Commun.* **25**, 8-13 (1966).
4. McKee, E.E., Ferguson, M., Bentley, A.T. & Marks, T.A. Inhibition of mammalian mitochondrial protein synthesis by oxazolidinones. *Antimicrob. Agents Chemother.* **50**, 2042-2049 (2006).
5. Moullan, N. *et al.* Tetracyclines disturb mitochondrial function across eukaryotic models: a call for caution in biomedical research. *Cell Rep.* (2015).
6. van den Bogert, C., Holtrop, M., Melis, T.E., Roefsema, P.R. & Kroon, A.M. Different effects of oxytetracycline and doxycycline on mitochondrial protein synthesis in rat liver after long-term treatment. *Biochem. Pharmacol.* **36**, 1555-1559 (1987).
7. Spooner, P.R. Oxytetracycline inhibition of mitochondrial protein synthesis in bovine lymphocytes infected with *Theileria parva* or stimulated by mitogen. *Parasitology* **101 Pt 3**, 387-393 (1990).
8. Terpstra, P. & Butow, R.A. The role of Var1 in the assembly of yeast mitochondrial ribosomes. *J. Biol. Chem.* **254**, 12662-12669 (1979).
9. Maheshwari, K.K. & Marzuki, S. Defective assembly of the mitochondrial ribosomes in yeast cells grown in the presence of mitochondrial protein synthesis inhibitors. *Biochim. Biophys. Acta* **824**, 273-283 (1985).
10. Fiorillo, M., Toth, F., Sotgia, F. & Lisanti, M.P. Doxycycline, Azithromycin and Vitamin C (DAV): A potent combination therapy for targeting mitochondria and eradicating cancer stem cells (CSCs). *Aging (Albany N. Y.)* **11**, 2202-2216 (2019).
11. Santo-Domingo, J., Chareyron, I., Broenimann, C., Lassueur, S. & Wiederkehr, A. Antibiotics induce mitonuclear protein imbalance but fail to inhibit respiration and nutrient activation in pancreatic β-cells. *Exp. Cell Res.* **357**, 170-180 (2017).
12. Zagorski, W. *et al.* Phenotypic suppression and nuclear accommodation of the mit$^-$ *oxi1*-V25 mutation in isolated yeast mitochondria. *Curr. Genet.* **12**, 305-310 (1987).

13. Dujardin, G., Lund, P. & Slonimski, P.P. The effect of paromomycin and [psi] on the suppression of mitochondrial mutations in *Saccharomyces cerevisiae*. *Curr. Genet.* **9**, 21-30 (1984).
14. Vargas Moller-Hergt, B., Carlstrom, A., Suhm, T. & Ott, M. Insertion defects of mitochondrially encoded proteins burden the mitochondrial quality control system. *Cells* **7**, 1-10 (2018).
15. Hobbie, S.N. *et al.* Genetic analysis of interactions with eukaryotic rRNA identify the mitoribosome as target in aminoglycoside ototoxicity. *Proc. Natl. Acad. Sci. U. S. A.* **105**, 20888-20893 (2008).
16. Davey, P.J., Haslam, J.M. & Linnane, A.W. Biogenesis of mitochondria. 12. The effects of aminoglycoside antibiotics on the mitochondrial and cytoplasmic protein-synthesizing systems of *Saccharomyces cerevisiae*. *Arch. Biochem. Biophys.* **136**, 54-64 (1970).
17. Jones, C.N., Miller, C., Tenenbaum, A., Spremulli, L.L. & Saada, A. Antibiotic effects on mitochondrial translation and in patients with mitochondrial translational defects. *Mitochondrion* **9**, 429-437 (2009).
18. Wilson, D.N. Ribosome-targeting antibiotics and mechanisms of bacterial resistance. *Nat. Rev. Microbiol.* **12**, 35-48 (2014).
19. Lin, J., Zhou, D., Steitz, T.A., Polikanov, Y.S. & Gagnon, M.G. Ribosome-targeting antibiotics: modes of action, mechanisms of resistance, and implications for drug design. *Annu. Rev. Biochem.* **87**, 451-478 (2018).
