# Supplementary material for: A stalled-ribosome rescue factor Pth3 is required for mitochondrial translation against antibiotics in Saccharomyces cerevisiae
Source: Commun Biol. 2021 Mar 8;4:300. doi: 10.1038/s42003-021-01835-6 (PMC7940416; doi:10.1038/s42003-021-01835-6)
Supplement: Supplementary file 3 — Description of Additional Supplementary Files [file 42003_2021_1835_MOESM3_ESM.pdf]

Description of Additional Supplementary Files

File name: Supplementary Data 1

Description: All source data underlying the graphs presented in the main figures.
